# Pediatric Blood Cancer Survivors and Tobacco Use across Adolescence and Emerging Adulthood: A Narrative Review

**DOI:** 10.3389/fpsyg.2016.00392

**Published:** 2016-03-21

**Authors:** Marianna Masiero, Silvia Riva, Chiara Fioretti, Gabriella Pravettoni

**Affiliations:** ^1^Department of Oncology and Hemato-Oncology, University of MilanMilan, Italy; ^2^Applied Research Division for Cognitive and Psychological Science, European Institute of OncologyMilano, Italy

**Keywords:** tobacco cigarette smoking, decision-making, pediatric blood cancer survivors, adolescence, emerging adulthood

## Abstract

Scholars underline the pivotal role of tobacco cigarette smoking in carcinogenesis process for blood tumors. A controversial debate is represented by the diffusion of tobacco use in young cancer survivors that had a previous diagnosis of blood tumor during the childhood. Compared with their peers, scientific evidence highlights that pediatric survivors have more difficult to give-up cigarette smoking. Furthermore, tobacco-smoking is frequently linked with others risk behaviors as drinking or substance abuse. In reviewing the main knowledge on this topic, authors affirm the need for increasing research on blood cancer survivors in order to depict psychological characteristics of pediatric blood cancer survivors. Improving health decision-making skills in young survivors could reduce the risk to adopt un-healthy behaviors and increase psychological wellbeing. Furthermore, authors propose tailored antismoking interventions based on the knowledge of the psychological and cognitive factors that support smoking during the transition toward emerging-adulthood.

## Introduction

Tobacco cigarette smoking has a pivotal role in carcinogenesis process for blood tumors, as well in surveillance for health related quality of life in survivors (U.S. Department of Health and Human Services, 2014). A controversial debate is represented by the diffusion of tobacco use in young cancer survivors that had a previous diagnosis of blood tumor during the childhood, as acute lymphoblastic leukemia (ALL) and acute myeloid leukemia (AML), considered the most common pediatric malignances. Broadly, pediatric survivors of blood tumors face with a high risk to incur in secondary tumors, and organ dysfunctions due to the received treatments; and cigarette smoking contributes to exacerbate these risks (Schultz et al., 2010). Even though researchers reported that overall cigarette smoking in leukemia survivors is lower than their siblings, they have similar probability to become regular smokers in long-term period. Literature on young adult survivors (defined by the literature as a subject who experiences a tumor diagnosis during the childhood; Langeveld et al., 2002) observed that the prevalence rates of tobacco use among pediatric survivors ranged from 8 to 29% (Tai et al., 2012). This suggested the “inefficacy” of the aforementioned cancer diagnosis to discourage pediatric survivors to consume cigarette (Kahalley et al., 2012).

These data are alarming, because pediatric survivors compared with their peers have more difficult to give-up, and cigarette-smoking is frequently linked with others risk behaviors as, drinking or substance abuse (Schultz et al., 2010; Tyc and Klosky, 2015).

Notwithstanding the perilous role of tobacco use for long-term health status of blood cancer survivors, currently studies are mainly focused on nutrition habits and physical activity, and merely borderline attention is given to smoking behavior, accepted for some studies (Emmons et al., 2003; Ford et al., 2014). These studies analyzed the role of the cigarette smoking behavior in young adult survivors considering various typology of cancer syndrome. No recent data, this topic are available (Tao et al., 1998) for pediatric survivors of blood tumors. In particular, a lack of clearness exists about the method used to evaluate smoking behavior in this specific group of cancer survivors, and the tendency to consider adult and young cancer survivors together (Kahalley et al., 2012). The majority of these studies are mainly focused on adult survivors, instead of adolescents and emerging adults.

## Adolescence and emerging adulthood: a psycho-cognitive root to understand tobacco behaviors

Despite, the critical clinical meaning for health related quality of life, poor educational interventions are developed to discourage cigarette smoking in young cancer survivors for blood tumors. Basically, developed projects aimed to improve health-protective behaviors or decision making strategies related to risk behaviors in adolescent survivors (Hollen et al., 2013). Anyway, not always interventions were effective and in none case they were focused on the specific behavior of smoking tobacco cigarette, and a significant knowledge lacking exists as young leukemia survivors. Indeed, all evidence available is referred to general cancer survivors, without focusing particularly on leukemia survivors, thus without taking into account the specific disease's characteristics and psychological need of this critical target population. Data available previously displayed that young adult survivor commonly initiated smoking at an earlier age as their peer, and the amount of cigarette increased during the time (Asfar et al., 2015). Tao et al. (1998), for instance, found that older age was predictive of increased likelihood to smoke regularly for both survivors and control subjects. This confirmed that early adolescence and emerging adulthood are two critical periods of the time both for smoking initiation and for continued smoking. Furthermore, no studies have also distinguished between adolescents and emerging adults survivors, considering the important lifespan transitions, and the specific developmental tasks that people have to acquire in the two different phases of life.

Studies in developmental psychology underline that adolescence and emerging adulthood are two important phases of the lifespan in which the individual develops some important identity changes and faces the emergence of risk behaviors. Although very chronologically close, these two phases have deeply specific developmental processes, as scholars underlined starting from many decades ago (Erikson, 1959; Arnett, 1997, 2000; Kroger and Marcia, 2011). While across adolescence kids are prone to monitor their identity status and to research their sense of Self (Erikson, 1959; Kroger and Marcia, 2011), emerging adults experience a sort of consolidation of their identity attempting to define who they are and which is their place in the world (Arnett, 2000). Furthermore, across the transition between adolescence and emerging adulthood some important cognitive processes develop (Habermas and Bluck, 2000; Fioretti and Smorti, 2015), improving young adults' ability to reflect on and elaborate past life experiences.

Besides, in this particularly stage of life individuals are more vulnerable to the cigarette smoke and its motivational properties (de la Peña et al., 2015). Mainly, leukemia survivors can feel during the changeover from adolescence to emerging-adulthood a double pressures: from the hand, the increasing of the anxiety and depression levels connected to the fear and worry for the disease recurrence; whereas from the other hand, they can engage in risk behaviors, to answer the need of self-identity determination, to the wish to be enrolled within the peer group, and to define their social status. Social status and positive outcome expected are considered a positive reinforcement for the smoking behavior. Sussman (2005) affirmed that at least cigarette smoking in young cancer survivors is positive related with perception that social consequences of tobacco use are positive, for example, psychological aspects as sensation seeking, environmental aspect as easy access to the cigarette (house, school, peer's group), cognition, and physiological reinforcement (a better attention level, more ability in problem-solving task and so on), and addictive properties of the nicotine (a robust vulnerability to the nicotine dependence; Sussman, 2005).

Also, the risk to incur in tobacco use is higher in leukemia survivors, who experienced a heavy psychological pain and emotional distress, who have experience of social exclusion and are involved in disadvantaged groups or ethnic minority, or live in household with others smoker (parents and/or siblings; Tyc and Klosky, 2015). Coherently, the family is an important modulation factor in cigarette smoking. It was reported that negative and stressor events within the family (as parental divorce) increase the risk to use cigarette smoking in adolescents survivor to an acute lymphoblastic leukemia diagnosis (Kahalley et al., 2012).

Frequently, cigarette smoking in young cancer survivors is associated with the practice of other risk behaviors. This sentence is confirmed by an investigation on 796 reported that in young cancer survivors cigarette smoking is matched with other maladaptive health behaviors as: low level of physical inactivity, exaggerated alcohol consumption, unhealthy diet, a low compliance with the vitamin therapy, and a general lack with the health care (Nathan et al., 2009). This implies pivotal implication for the general health-status, because subjects increase seriously their risk of recurrence combing multiple risk factors (Tai et al., 2012).

Some Authors suggested that the attitude to adopt unhealthy behaviors may be explained used the psycho-cognitive construct of the optimistic bias (Weinstein and Lyon, 1999). This cognitive prejudice pushes the subject to undervalue the risk when concern himself, while he tends to change his attitude when the risk concerns other people. This cognitive bias is particular strong in smokers and young people (Lucchiari et al., 2015). The optimistic bias drives to develop “false beliefs” to be protected by negative risks related to the cigarette. Ford and colleagues in a study of young cancer patient (leukemia, Hodgkin's disease, Non-Hodgkin's lymphoma, CNS malignancy, Bone cancer) observed that 20% did not recognized the positive correlation between cigarette consumption and their risk to increase health hazards in the next future (Ford et al., 2014). Besides, a higher perception of vulnerability to incur in smoking related disease or cancer relapse influences the ability to interrupt or to limit tobacco exposure (Emmons et al., 2003).

## Decision making skills and blood tumor treatments: the impact on risk behaviors

Another critical point is that the childhood cancer survivors' knowledge of their diagnosis and treatment is often inadequate, as well as their knowledge level about the risks of medical late-effects (Syed et al., 2015). Merely 15% of childhood and adolescent cancer survivors have received by the physicians a comprehensive treatment and survivorship care plan. So, they are uninformed about the long-term risks of their childhood tumor (Ford et al., 2014). These factors dramatically contribute to decrease decision-making about the adoption of health protective behaviors. Concerning to the last point, Hollen et al. (2013) argued that the high percentage of risk behavior in cancer survivors is due to poor decision-making skills. On the whole, young survivors of acute lymphoblastic leukemia are exposed to side-effects of the antineoplastic therapies that can contribute to damage the central executive functions. This “cognitive impairment” is might be considered as a prognostic factor of the smoking in the next future, because it reduces decision-making ability and increases risk-taking. Coherently with these data, it was highlighted that the intensive medical treatments exposition is positive correlated to the risk to be involved in health risk behaviors during the adulthood (Li et al., 2015). Authors supposed that this positive association is explained by a lower education (Bauld et al., 2005). Particularly, young cancer patients spend a long period of time in hospital for the treatments during the childhood, so they may experience during adolescence (secondary high school) and emerging adult (transition from university to work) social and school failure.

Indeed, it is well-known that young leukemia survivors frequently experience specific education need and/or learning difficult (Pi, 1999). As cited above, learning difficulty and a general impairment of the cognitive processing can be affected by the treatments received during the childhood that caused neuropsychological problems, in different areas as short term memory, and visual motor coordination, that they can decreasing learning skills. This has as principal side-effect a low educational level comparing to their peer, and overall, a more difficult in social relation (Tai et al., 2012). Thus, a low educational level will have as a consequence a high probability to be unemployed in the next future. A risk factor for smoking in young cancer survivors is considered “to be unemployed” (e.g., Langeveld et al., 2002; Asfar et al., 2015). In a study on 580 young adult survivor with a childhood diagnosis of acute lymphoblastic leukemia, it was reported that comparing young adult survivors vs. sibling controls were jobless or were working less than half-time (Zeltzer et al., 1997 in Langeveld et al., 2002). Authors think that the impossibility to work has a negative impact on emotional disposition, increasing negative affect and depression mood. Despite to this, there are not general agreements in scientific literature (Langeveld et al., 2002).

## Liquid vs. solid tumors: a different risk perception prospective

Likewise, the studies conducted on young cancer survivors neglect the specificity of the blood tumors, that it has important psychological root. One characteristic of blood tumors is that it is not possible to “limit the disease” to a particular region of the body as the entire organism is affected by the disease. Some authors reported that differently from patients with a solid tumor (e.g., breast and prostate tumor), blood tumors patients, have more troubles understanding, and managing health information and health behavior (Riva et al., 2015; Riva and Pravettoni, 2016). This difficulty is strictly related to the unfeasibility to detect a definite disease's site (Baccarani et al., 2014).

Managing a disease where the body localization is not easy to define appear to be more troublesome because the person feels to have less control or disease monitoring over the time (Renzi et al., 2015). This inability to detect tumor site can increase fear and worry for the disease development. It has been recently identified that adolescent with blood tumors have more emotional problems like anxiety, depression, and posttraumatic stress disorder than other and these problems may get more difficult the management of the condition and a good health status (Muffly et al., 2016). Notwithstanding the key importance for patient care involvement, few evidence is available on this topic. We hypothesize that the difficult to mentally represent the “cancer localization” may contrast with the comprehension and adoption of better lifestyles.

## Future perspectives

Basing on these assumptions, we point out some pivotal considerations.

First, epidemiological data reported a growing of five-year survival rates for young cancer patients with leukemia, from 83.7% in 1990–1994 to 90.4% in 2000–2005, while 10-years survival was increased from 80.1% between 1990 and 1994 to 83.9% in 1995–1999. Data showed likewise that ~84% of deaths occurred within 5 years from the diagnosis, whereas only 1% occurred 10 years later after the diagnosis (Hunger et al., 2012). Thus, a high proportion of subjects experienced a hematological malignancy in childhood, while in first adolescence face almost 5 years of periodic follow-ups, attending medical interviews and clinical examinations across such delicate lifespan transitions as adolescence and emerging adulthood are. Considering the high percentage of this cancer in pediatric stage and the consequences in long time period, it is important increasing research on blood cancer survivors in order to depict psychological characteristics and needs of this population (Cropley et al., 2008). This might provide indispensable information about how to improve health decision-making skills in young survivors, reducing the risk to adopt un-healthy behaviors and increasing psychological wellbeing.

Second, as cited above the adolescence and emerging adulthood are two very close, but different periods concerning identity development and risk attitudes. In this sense, smoking attitudes change across the lifespan and interventions (such as educational activities at school, health campaigns at hospitals' departments) on them should be implemented considering the different age of participants.

Third connected to the previous points, the-long term follow-ups provide the opportunity for the oncologists to support and educate young survivors to adopt no-smoking behaviors. These goals might be achieved only promoting the implementation of tailored antismoking interventions based on the knowledge of the psychological and cognitive factors that support smoking during the transition toward emerging-adulthood. This kind of intervention is coherent with the personalized medicine model suggested in previous researches on cancer patients (Kondylakis et al., 2012, 2013; Cutica et al., 2014). A tailored approach should be based on a psycho-emotional clinical assessment adapted to the characteristics of the cancer survivors. Also, it should be associated with a personal feedback by health professionals and tailored health materials (booklet, video, and interactive tools, APP) usable to support smoking cessation or to avoid initiation. We presume that this model might increase the coping strategies and the decision-making abilities that are considered milestones to improve non-smoking behaviors in this stage of life (Sussman, 2013). Concluding, we argue that this tailored approach to the treatment of cigarette smoking behavior in pediatric blood cancer survivors will improve their health related quality of life.

## Author contributions

The review was conceived by MM and SR. Data extraction was carried out by MM, SR, and CF with support from GP. Reporting of findings was led by MM with support from SR and CF. All authors contributed to manuscript preparation and approved the final version.

### Conflict of interest statement

The authors declare that the research was conducted in the absence of any commercial or financial relationships that could be construed as a potential conflict of interest.
